# Bioactive Molecules from Plants: Discovery and Pharmaceutical Applications

**DOI:** 10.3390/pharmaceutics14102116

**Published:** 2022-10-05

**Authors:** Maria Camilla Bergonzi, Charles M. Heard, Javier Garcia-Pardo

**Affiliations:** 1 Department of Chemistry, University of Florence, Via Ugo Schiff 6, Sesto Fiorentino, 50019 Florence, Italy; 2 School of Pharmacy and Pharmaceutical Sciences, Cardiff University, Cardiff CF10 3NB, UK; 3 Institut de Biotecnologia i Biomedicina and Departament de Bioquimica i Biologia Molecular, Universitat Autònoma de Barcelona, Bellaterra, 08193 Barcelona, Spain

The plant kingdom is one of the richest sources of bioactive compounds with pharmaceutical potential. A special feature of higher plants is their ability to produce a great number of secondary metabolites of a high chemical diversity. Plants are also enriched in wide variety of diverse proteins, peptides, sugars and nucleosides, which are often involved in plant primary physiological functions and/or in mechanisms against pathogens. These compounds can be widely found in fruits, seeds, flowers and leaves, from where they can be extracted and purified by different techniques. For decades, a plethora of bioactivities have been found in molecules isolated from plants, and they are still one of the leading sources of novel bioactive compounds. This editorial aims to summarize the 33 articles (27 research articles, 3 communications and 3 reviews) that contributed to the first edition of this Special Issue ‘Bioactive Molecules from Plants: Discovery and Pharmaceutical Applications’. The original research contributions included herein address the identification and characterization of novel molecules with a wide spectrum of biological activities. The Special Issue also includes several original reports describing the encapsulation and delivery of plant-derived compounds. In the following sections, we review all the articles in our Special Issue, and we believe this editorial will be of interest to a broad readership in the field.

Many plant-derived compounds have been used in the past because of their antimicrobial properties. Four studies in this Special Issue report the characterization of novel bioactive molecules and plant-derived biopolymers with strong antimicrobial properties [1,2,3,4]. Plants are also an important source of antioxidant peptides. In this volume, three publications investigated the antioxidant and ROS scavenging properties of plant-derived molecules [1,5,6]. Two of these studies by Cotabarren et al. have also shown the isolation and characterization of thermostable peptides with anticoagulant properties [2,6]. In addition, the study of Lee et al. has shown the identification of a bioactive natural product with potential anti-inflammatory activity [7]. The article from Tavares et al. studied the effect of the essential oil of *Cymbopogon winterianus*, a natural product with antioxidant, anti-inflammatory and antifibrotic properties, in the progression of histological changes of pulmonary fibrosis in a rodent model [8]. The article of Abdalla et al. drives us more into the importance of plant nutrition. They applied metabolomic approaches to investigate the effects of sulfur nutrition on the metabolic profiles and biological activities of three different *Lactuca sativa* (lettuce) cultivars [9].

Probably plants are the most well-known source of molecules with demonstrated anticancer therapeutic properties. Another set of papers of this Special Issue have investigated the antitumoral properties of plant extracts and plant-derived compounds, revealing interesting antiproliferative and/or pro-apoptotic activities in vitro [10,11,12,13,14,15,16,17,18]. In addition, Huang and coworkers have shown that Ceylon olive leaf extract and its phytochemicals, such as mearnsetin, displayed potent antimelanogenesis effects in a zebrafish model [19]. Many of these studies have provided key mechanistic insights into their specific biological functions.

In this Special Issue, we also expanded our knowledge on plant-derived compounds with a role in modulating key targeted cellular signaling pathways. An interesting study by Bertozzi and coworkers has shown that taro lectin can promote immunomodulatory effects by acting as a cytokine-mimetic compound [20]. On the other hand, Lee and coworkers reported that schisandrol A exhibits estrogen-like properties by promoting estrogenic activity via the estrogen receptor α-dependent signaling pathway [21]. Additionally, two studies have been conducted to investigate the antidiabetic properties of naturally occurring molecules from plants. The first work by Caroleo et al. showed that olive oil lipophenols can induce insulin secretion in an in vitro β-cell model [22]. The second study led by Lim et al. suggests that two flavonoids isolated from *Morus alba* fruits have the potential to promote insulin-stimulated glucose uptake in 3T3-L1 adipocytes [23]. Together, these two works indicate that bioactive molecules from plants can be potentially exploited for glycemic regulation in the management of type 2 diabetes mellitus (T2DM).

Increasing evidence indicates that compounds derived from some plants have a role in neuroprotection. Two articles of this Special Issue focused on the utilization of plant-derived compounds as neuroprotective [24] or neuromodulatory [15] agents. In the first of such studies, Meganova and coworkers evaluated the effects of novel hydroxamic acid derivatives in a 5xFAD transgenic mice model of Alzheimer’s disease (AD). Notably, one of the leading compounds was able to restore normal memory functions to the level observed in control wild-type animals [24]. In the second study, contributed by De Oliveira et al. to this Special Issue, the authors investigated the effects of saffron extract pre-treatment for prevention of stress-induced depressive-like behaviors [25]. Taken together, these studies highlight the potential of compounds derived from plants for the prevention and treatment of AD and other neurodegenerative diseases.

In the last couple of decades, numerous methods for the encapsulation of plant-derived molecules have been developed for drug delivery purposes. It has been increasingly recognized that this approach is useful to improve the solubility, biodistribution and successful delivery of naturally occurring molecules. This Special Issue also includes selected contributions addressing this topic. For example, Landucci et al. reported the fabrication of two liposomal formulations developed for the efficient encapsulation of thymoquinone, a phytochemical compound found in the seeds of *Nigella sativa*. They demonstrate the efficacy of these preparations, achieving improved ocular delivery of this compound [26]. Moreover, a publication by Magalhães et al. has shown that the essential oil nanoemulsion substantially improved its inhibitory activity [27]. Hydrogels also appear to be an excellent approach for controlled drug delivery. Along this line, Batista et al. presented a new thermosensitive hydrogel topical formulation containing *Viscum album* (mistletoe) extracts [28]. An additional publication explored the incorporation of cyclodextrin during resveratrol extraction. By using this approach, the authors improved resveratrol solubility, which at the same time enhanced mucoadhesive properties of the final formulation [29]. In the same direction, a comprehensive study by Kobryń and coworkers investigated the optimal solvent conditions for dermal hydrogel formulations with cryptotanshinone [30].

The last three contributions of this Special Issue constitute a collection of high-quality updated reviews. The first review written by Lupaescu et al. covers the role of various small bioactive molecules known to ameliorate amyloidosis [31]. They also discuss the link between these compounds and the prevention and development of T2DM and AD. The second review contributed by Filipiuc et al. dissects current findings on phytocannabinoids, including a detailed description of their cannabinoid and non-cannabinoid receptors [32]. The third review submitted by Manganyi et al. is a critical review summarizing current roles of therapeutic and toxic compounds derived from *Drimia* species [33].

In conclusion, the publications in this research topic highlight the diverse current efforts in identifying novel molecules derived from plants, providing a detailed view of the field, from compound identification to their therapeutic use.
